# Canine osteosarcoma cells exhibit resistance to aurora kinase inhibitors

**DOI:** 10.1186/1753-6561-7-S2-P15

**Published:** 2013-04-04

**Authors:** Claire M Cannon, John Pozniak, Milcah C Scott, Daisuke Ito, Brandi H Gorden, Ashley J Graef, Jaime F Modiano

**Affiliations:** 1 Department of Veterinary Clinical Sciences, College of Veterinary Medicine, University of Minnesota, St Paul, MN, USA; 2 Masonic Cancer Center, University of Minnesota, MN, USA

## Background

Aurora kinases are overexpressed and associated with poor prognosis in several human tumours. Recently, overexpression and correlation with poor prognosis was found in canine osteosarcoma. We examined the effects of Aurora kinase inhibition in canine osteosarcoma cell lines. This was the first step to explore development of Aurora kinase inhibitors (AKIs) as novel therapeutic agents for canine osteosarcoma.

## Materials and methods

We evaluated the effects of two AKIs, AZD1152 and VX680, on four canine osteosarcoma cell lines. Viability was assessed using the MTS assay, apoptosis using caspase activation and flow cytometry for Annexin V binding and 7-AAD uptake, and target modulation using Western blotting. We also evaluated gene and protein expression of Aurora kinase B, ABCB1 and ABG2.

## Results

Expression of Aurora kinase B protein and mRNA was seen in all cell lines. Although Aurora kinase inhibitors induced cytotoxicity, half-maximal inhibitory concentrations were significantly higher than seen in other cancer types and induction of apoptosis was minimal at concentrations close to IC50s. AZD1152 reduced Aurora kinase B phosphorylation, indicating effective target modulation. ABCB1 and ABCG2 transporter-mediated efflux is one known mechanism of resistance against these drugs. Verapamil modestly enhanced AZD1152-mediated apoptosis and ABC transporters were expressed by a small percentage of cells, suggesting that other mechanisms may contribute to resistance.

## Conclusions

Canine osteosarcoma cells are resistant to AKIs and we suggest that these compounds are unlikely to be useful as monotherapy for this disease. Further investigation of resistance mechanisms and the potential utility of AKIs in combination therapy is warranted.

